# Kir3 channel signaling complexes: focus on opioid receptor signaling

**DOI:** 10.3389/fncel.2014.00186

**Published:** 2014-07-08

**Authors:** Karim Nagi, Graciela Pineyro

**Affiliations:** ^1^Département de Pharmacologie, Faculté de Médecine, Université de MontréalMontreal, QC, Canada; ^2^Centre de Recherche du CHU Sainte-JustineMontréal, QC, Canada; ^3^Département de Psychiatrie, Faculté de Médecine, Université de MontréalMontréal, QC, Canada

**Keywords:** GIRK, opioid, signaling, analgesia, bias, signaling complexes, functional selectivity, allosteric modulation

## Abstract

Opioids are among the most effective drugs to treat severe pain. They produce their analgesic actions by specifically activating opioid receptors located along the pain perception pathway where they inhibit the flow of nociceptive information. This inhibition is partly accomplished by activation of hyperpolarizing G protein-coupled inwardly-rectifying potassium (GIRK or Kir3) channels. Kir3 channels control cellular excitability in the central nervous system and in the heart and, because of their ubiquitous distribution, they mediate the effects of a large range of hormones and neurotransmitters which, upon activation of corresponding G protein-coupled receptors (GPCRs) lead to channel opening. Here we analyze GPCR signaling via these effectors in reference to precoupling and collision models. Existing knowledge on signaling bias is discussed in relation to these models as a means of developing strategies to produce novel opioid analgesics with an improved side effects profile.

Opioids produce their actions via the activation of μ (MOR), δ (DOR), and κ (KOR) receptors (Pert and Snyder, 1973; Simon et al., 1973) all of which couple to heterotrimeric Gi/o proteins. As such they share adenylyl cyclase, Phospholipase Cβ [PLCβ, N-type Ca^2+^ (Cav2.2)] channels and G-protein coupled inward rectifier K^+^ channels (GIRKs or Kir3 channels) as canonical Gα and Gβγ effectors. The two channel effectors prominently mediate analgesic effects of opioids. For example, studies in rodents have shown that activation of MORs on small, unmyelinated nociceptors (Scherrer et al., 2009; Heinke et al., 2011; Bardoni et al., 2014) contribute to the analgesic effects of morphine (Wei et al., 1996; Andrade et al., 2010), and this is done via inhibition of N-type Ca+2 channels that control neurotransmitter release from these primary afferents (Glaum et al., 1994; Wrigley et al., 2010). Kir3 channel contribution to opioid analgesia has also been demonstrated in animal models (Ikeda et al., 2000; Mitrovic et al., 2003; Marker et al., 2005), and their relevance in the clinical response to opioid analgesics has been further confirmed by gene linkage analyses (Nishizawa et al., 2009; Lotsch et al., 2010; Bruehl et al., 2013). In addition, the use of genetically modified mice lacking different channel subunits has further established activation of Kir3 channels as a pervasive analgesic mechanism which, in addition to opioids, also mediates pain modulation by α2 adrenergic, muscarinic, GABA_B_ and cannabinoid receptor ligands (Blednov et al., 2003; Mitrovic et al., 2003). From this perspective the development of direct Kir3 channel activators may be considered a promising strategy for the development of novel analgesics. However, enthusiasm for this particular approach has remained limited given the possibility of widespread side-effects associated with administration of direct channel openers (Lujan et al., 2014). Indeed, these would result from the great variety of physiological responses mediated by Kir3 channels, including heart frequency control (Wickman et al., 1998; Bettahi et al., 2002), memory (Wickman et al., 2000), learning (Wickman et al., 2000; Cooper et al., 2012), as well as their participation in different pathological conditions including development of seizures (Signorini et al., 1997), generation of anxiety states (Blednov et al., 2001; Pravetoni and Wickman, 2008) and contribution to abnormal plasticity by drugs of abuse (Padgett et al., 2012; Hearing et al., 2013). Consequently, the prevailing strategy for engaging Kir3-mediated analgesia has remained the activation of pain-modulating GPCRs (McAllister et al., 1999; Lujan et al., 2014). Among them MORs, DORs and KORs all three mediate effective analgesia but display different side effects that distinctively limit their therapeutic use (Kieffer and Gaveriaux-Ruff, 2002; Bruchas and Chavkin, 2010; Gaveriaux-Ruff and Kieffer, 2011). Here we will summarize Kir3 channel contribution to desired and undesired responses of opioid analgesics and address the question of whether biasing opioid signaling toward these effectors could help develop better opioid analgesics with a reduced side effects profile.

## Kir3 channel signaling and opioid-dependent analgesia

Kir3 channels are formed by tetrameric association of four different subunits (Kir3.1-3.4). Although all four of them are expressed in peripheral (Gao et al., 2007) and central nervous system (Wickman et al., 2000; Saenz Del Burgo et al., 2008; Fernandez-Alacid et al., 2011), neural channels are most frequently formed by association of Kir3.1, 3.2 and 3.3 subunits (Liao et al., 1996; Hibino et al., 2010; Luscher and Slesinger, 2010). The subunit combination that forms the channel will depend on the neuronal population involved (Inanobe et al., 1999; Cruz et al., 2004) and the cellular compartment (Koyrakh et al., 2005; Fernandez-Alacid et al., 2009) in which channels are found. Nonetheless, Kir3.1/3.2 heterotetramers are considered prototypical neuronal channels.

Kir3 channel contribution to opioid analgesia was initially suggested by the fact that mice carrying a missense mutation which renders Kir3.2 subunits insensitive to G protein activation (Patil et al., 1995; Navarro et al., 1996) displayed reduced analgesic responses to morphine (Ikeda et al., 2000, 2002). These observations were subsequently confirmed using null mice for different subunits (Mitrovic et al., 2003; Marker et al., 2005), which allowed to also implicate Kir3.1 (Marker et al., 2004) and Kir3.3 (Marker et al., 2002) in pain modulation. Knock-out of Kir3.2 and 3.3 was shown to interfere with morphine's ability to prolong avoidance behavior in the hot plate test. Since this response involves supraspinal integration, it can be concluded that both subunits contribute to mechanisms of opioid analgesia at this level (Marker et al., 2002; Mitrovic et al., 2003). Possible sites of supraspinal Kir3-mediated analgesia may include thalamus and limbic cortex, both of which express Kir3.1, 3.2 and 3.3 subunits (Saenz Del Burgo et al., 2008; Fernandez-Alacid et al., 2011) as well as opioid receptors (Le Merrer et al., 2009). The midbrain periaqueductal gray seems less likely since in this nucleus opioid actions are primarily presynaptic reducing neurotransmitter release via a mechanism that involves phospholipase A2, arachidonic acid and 12-lipoxygenase, which leads to modulation of voltage-dependent potassium channels (Vaughan and Christie, 1997; Vaughan et al., 1997).

Genetically engineered mice lacking Kir3.1 or Ki3.2 subunits also display reduced responses to intrathecal administration of morphine in the tail flick test (Marker et al., 2004, 2005) implicating both subunits in spinal mechanisms of opioid analgesia. This interpretation is supported by reports locating Kir3.1 and Kir3.2 subunits to lamina II interneurons that co-express μ opioid receptors (Marker et al., 2006). Functional studies also indicated that silencing of Kir3.1 or Kir3.2 subunits, or intrathecal infusion of Kir3 channel blocker tertiapin-Q interfered with the analgesic response by MOR and DOR agonists administered by the same route (Marker et al., 2005). In contrast, Kir3.3 ablation was without effect on the analgesic response elicited by intrathecal morphine (Marker et al., 2004), arguing against its significant contribution at this level. The latter observation is also in keeping with immunohistological studies which reported absence of Kir3.3 labeling in the dorsal horn (Marker et al., 2004).

In addition to their participation in acute opioid analgesia, spinal Kir3 channels seem to mediate neuroadaptations that modulate responsiveness to opioids in conditions such as inflammatory or cancer-related pain. This possibility is particularly suggested by reports indicating that carrageenan-inflammation (Gonzalez-Rodriguez et al., 2010) and bone cancer (Gonzalez-Rodriguez et al., 2012) enhance the inhibitory effect of Kir3 channel blocker tertiapin-Q on analgesia induced by intrathecal administration of morphine.

Apart from brain and spinal cord, opioid receptors are also present in sensory neurons of the dorsal root ganglion (DRG) (Li et al., 1998; Gendron et al., 2006; Wu et al., 2008; Wang et al., 2010) and are transported to peripheral terminals where they mediate analgesic actions of peripherally injected opioids (Hassan et al., 1993; Obara et al., 2009; Vadivelu et al., 2011). Transcripts for Kir3.1/3.2 subunits have been detected in human DRGs and in rat nociceptors, but not in mice (Gao et al., 2007; Nockemann et al., 2013). This distribution parallels differences in species sensitivity to peripheral administration of opioids suggesting that when expressed, DRG Kir3 subunits actively participate in opioid analgesia. Indeed, intraplantar injection of MOR agonists does not produce analgesia in mice (Nockemann et al., 2013) but effectively mitigates pain in inflammatory or neuropathic rat models (Stein et al., 1989; Obara et al., 2009; Chung et al., 2013; Nockemann et al., 2013) as well as postoperative and arthritic pain in humans (Kalso et al., 2004; Vadivelu et al., 2011). Moreover, the active contribution of Kir3.2 channels to peripheral opioid analgesia has now been experimentally established using transgenic mice genetically engineered to express these subunits in sensory neurons. Unlike their wild-type counterparts, the latter display an analgesic response to plantar application of MOR agonist DAMGO (Nockemann et al., 2013).

It is important to bear in mind that most of the evidence analyzed thus far links Kir3 channel function to opioid analgesia in animal models. However, the latter have shown somewhat limited success in identifying and validating analgesic targets of clinical relevance (Mogil, 2009). Hence, from this perspective, any evidence linking Kir3 channels to therapeutic response in humans is of specific interest. At least three studies have now shown that variations in the gene coding for Kir3.2 subunits (*KCNJ6*) influence opioid dose requirements for both acute management of postoperative pain (Nishizawa et al., 2009; Bruehl et al., 2013) and for pain control in chronic patients (Lotsch et al., 2010). Similar assessment of Kir3.1 (*KCNJ3*) variations showed no effect (Bruehl et al., 2013).

## Opioid side effects and Kir3 channel signaling

In spite of the established analgesic efficacy of MOR, DOR, and KOR agonists, activation of the different receptor subtypes results in a distinct set of side effects which limit therapeutic application of their agonists. Constipation, nausea, respiratory depression, tolerance, dependence, and abuse are among the most common undesired effects of clinically available MOR agonists (Ballantyne and Shin, 2008; Morgan and Christie, 2011). Many of these undesired actions, particularly respiratory depression (Cheng et al., 1993; Gallantine and Meert, 2005), gastrointestinal side effects (Tavani et al., 1990; Gallantine and Meert, 2005; Feng et al., 2006) and physical dependence (Cowan et al., 1988; Codd et al., 2009) are less severe or absent with DOR agonists. Moreover, although DORs and MORs are both involved in reward response to physiological stimuli (Charbogne et al., 2014), DORs are not directly involved in assigning reward value to stimuli but instead facilitate predictive learning and influence the choice of action in face of contingencies that involve reward (Laurent et al., 2012). In keeping with this functional profile, DOR agonists do not facilitate intracranial self-stimulation (Do Carmo et al., 2009), are not discriminated as morphine substitutes (Gallantine and Meert, 2005) and do not display reinforcing properties in non-human primates (Banks et al., 2011). Nonetheless, despite these advantages, potential for tolerance (Pradhan et al., 2010; Audet et al., 2012) remains a limitation for long-term use of DOR agonists, as does their propensity to produce hippocampal hyperexcitability that may lead to seizures. The latter are produced both by DOR (De Sarro et al., 1992; Broom et al., 2002; Jutkiewicz et al., 2005) and MOR agonists (Drake et al., 2007). In the specific case of DOR agonists, hyperexcitability is brief and non-lethal. Moreover, seizures are rare at analgesic doses (Jutkiewicz et al., 2006), they do not appear with all ligands (Le Bourdonnec et al., 2008; Saitoh et al., 2011) and can be suppressed by controlling the rate of agonist administration (Jutkiewicz et al., 2005). Thus, overall, side effects of DORs agonists are considerably milder as compared to those of MORs, and their proven efficacy in chronic pain management make DORs especially attractive as targets for the development of novel analgesics (Gaveriaux-Ruff and Kieffer, 2011). This notion is further reinforced by the fact that DOR agonists display antidepressant properties (Chu Sin Chung and Kieffer, 2013) which could be of additional benefit in controlling negative affect, frequently associated with prolonged pain syndromes (Goldenberg, 2010). Unlike DOR agonists, KOR-mediated analgesia is typically associated with stress, depression, and dysphoria (Bruchas and Chavkin, 2010; Van't Veer and Carlezon, 2013) which, together with their tendency to induce tolerance (McLaughlin et al., 2004; Xu et al., 2004), would make these ligands less attractive for the treatment of chronic pain.

Kir3 channel function has been associated with some of the undesired effects of opioid analgesics. For example, reduced GABAergic activity in hippocampal dentate gyrus increases the excitability of glutamatergic granule cells and may reduce the threshold for seizures (Drake et al., 2007). MORs and DORs agonists silence hippocampal GABAergic interneurons by a mechanism that involves both Kir3 (Luscher et al., 1997) and voltage-dependent K^+^ channels (Wimpey and Chavkin, 1991; Moore et al., 1994), explaining their documented tendency to produce seizures (Drake et al., 2007). MORs agonists also enhance excitability and firing activity of dopaminergic neurons of the ventral tegmental area (Gysling and Wang, 1983), and do so by hyperpolarization of local interneurons (Johnson and North, 1992; Bonci and Williams, 1997). Like in hippocampus, disinhibition is mediated through Kir3 channel activation (Luscher et al., 1997) and its consequence is the enhanced release of dopamine in corticolimbic areas which is thought to facilitate compulsive behaviors characteristic of addiction (Luscher and Ungless, 2006). Finally, Kir3 channel activation by KORs modulates firing activity of dorsal raphe 5-HT neurons (Bruchas et al., 2007; Lemos et al., 2012). Dynorphin release during repeated stress exposure produces sustained activation of these receptors driving p38α-MAPK activity and subsequent Kir3.1 subunit phosphorylation (Lemos et al., 2012). As a consequence 5-HT neuron firing activity becomes deregulated, possibly contributing to dysphoric effects of uncontrollable stress and to aversive actions of KOR agonists (Bruchas et al., 2007).

Additional undesired actions of opioids analgesics involve signaling effectors other than Kir3 channels. For example, constipation and respiratory depression have been associated with β arrestin (β arr)-mediated signaling by MOR agonists (Raehal et al., 2005; Dewire et al., 2013). The use of β arr-knockout mice has also suggested that DOR agonist tendency to induce seizures may involve this regulatory protein, and biased agonists that fail to recruit β arrs are being developed as a means of further improving the side effects profile of analgesics acting at this receptor subtype. Kir3-independent side effects also include compensatory changes in the cyclase pathway (cyclase superactivation). These are triggered by sustained inhibition of cAMP production (Christie, 2008) and have been shown to contribute to physical dependence (Han et al., 2006; Cao et al., 2010; Yang et al., 2014) and analgesic tolerance of opioid agonists (Javed et al., 2004; He and Whistler, 2007; Bobeck et al., 2014).

Data summarized thus far indicate that undesired actions of opioid ligands segregate according to receptor subtype, and within each subtype, desired and unwanted effects are not all necessarily mediated by the same effectors. Thus, undesired actions of DOR and KOR-activating analgesics are less than those of MOR ligands, and among the former, the presence of antidepressant properties and lack of dysphoric actions makes DOR specifically interesting as putative targets for the management of chronic pain syndromes (Gaveriaux-Ruff and Kieffer, 2011; Pradhan et al., 2011). Tolerance however, remains a drawback that limits further development of DOR-acting analgesics. This limitation could be, at least, partly addressed by taking advantage of biased signaling since effectors that mediate cellular tolerance and analgesia seem partly segregated. Indeed, as detailed above, while analgesic actions are generally mediated via modulation of channel effectors, adaptations of the cAMP cascade seem to account for at least some of the manifestations of tolerance (Javed et al., 2004; He and Whistler, 2007; Bobeck et al., 2014). Based on these observations, opioid ligands that specifically bias their pharmacological stimulus toward channel effectors could conceivably conserve analgesic properties while displaying reduced potential for tolerance, particularly the component that depends on cyclase adaptations. In the following sections, we will consider possible strategies to direct opioid signaling toward Kir3 channels. However, before doing so it is worth revising the events that lead to channel activation.

## Kir3 channel activation via GPCRs

It is now well established that Kir3 channels open via direct interaction with Gβγ dimers that are released from pertussis toxin sensitive heterotrimeric Gi/o proteins upon receptor activation (Logothetis et al., 1987; Wickman et al., 1994; Raveh et al., 2009). Gβγ activates the channel as long as the surface that contacts Kir3 subunits does not re-associate with Gα_i/o_-GDP, which results in signal termination (Schreibmayer et al., 1996). Biochemical, mutational and nuclear magnetic resonance studies have mapped several interaction sites for Gβγ on all four channel subunits. These sites are summarized in Table 1 and those in the C-terminal cytosolic domain have been largely confirmed in recent crystallization of a complex formed by Gβ 1γ2 and the Kir3.2 homotetramer (Whorton and MacKinnon, 2013). Crystals of the complex revealed that the contact area between Gβ and the channel is approximately 700A°^2^. The contact zone on channel subunits corresponds to the interface of two contiguous cytosolic domains encompassing β sheets/loops βK, βL, βM, and βN on one subunit and βD-βE elements from the adjacent one. The channel's interaction surface on Gβ overlaps the Gα binding site (Ford et al., 1998; Whorton and MacKinnon, 2013). The arrangement of Kir3.2 and Gβγ subunits within the crystal corresponds to a membrane-delimited signaling complex consisting of one channel tetramer, four Gβγ subunits, four phosphatidylinositol 4,5-bisphosphate (PIP_2_) molecules and four Na^+^ ions bound to corresponding regulatory sites on the channel (Whorton and MacKinnon, 2011). This organization is compatible with Gβγ binding to the channel to induce an intermediate active state that is stabilized into the full open conformation by PIP_2_ and Na^+^ ions (Whorton and MacKinnon, 2011, 2013). Interaction sites for Gα_i/o_ have also been mapped to all four channel subunits, both in GDP- and GTP-bound states and these sites as summarized in Table 2.

**Table 1:**
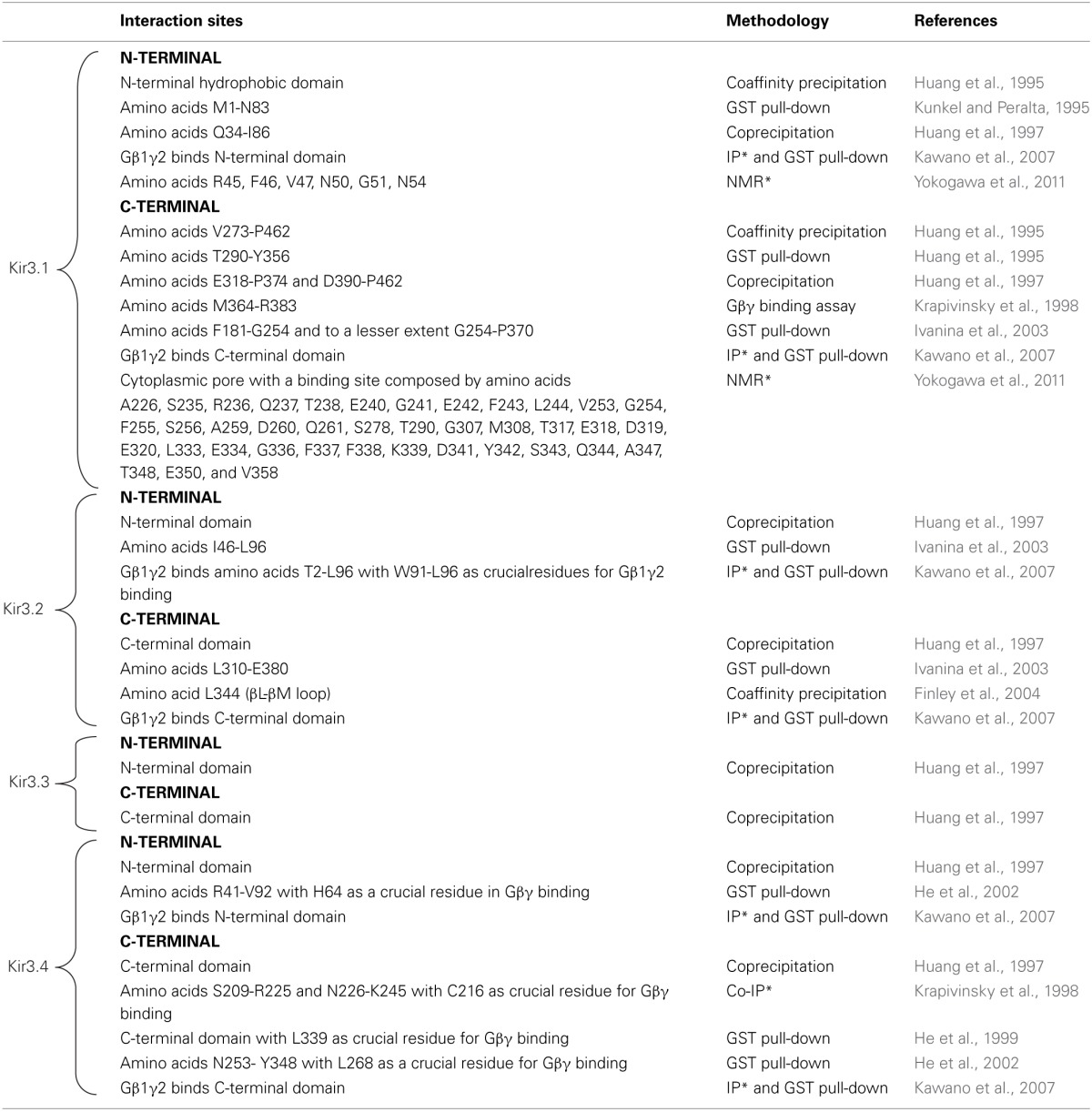
**Gβγ interaction sites on different Kir3 subunits**.

*IP, Immunoprecipitation; NMR, Nuclear magnetic resonance spectroscopy*.

**Table 2:**
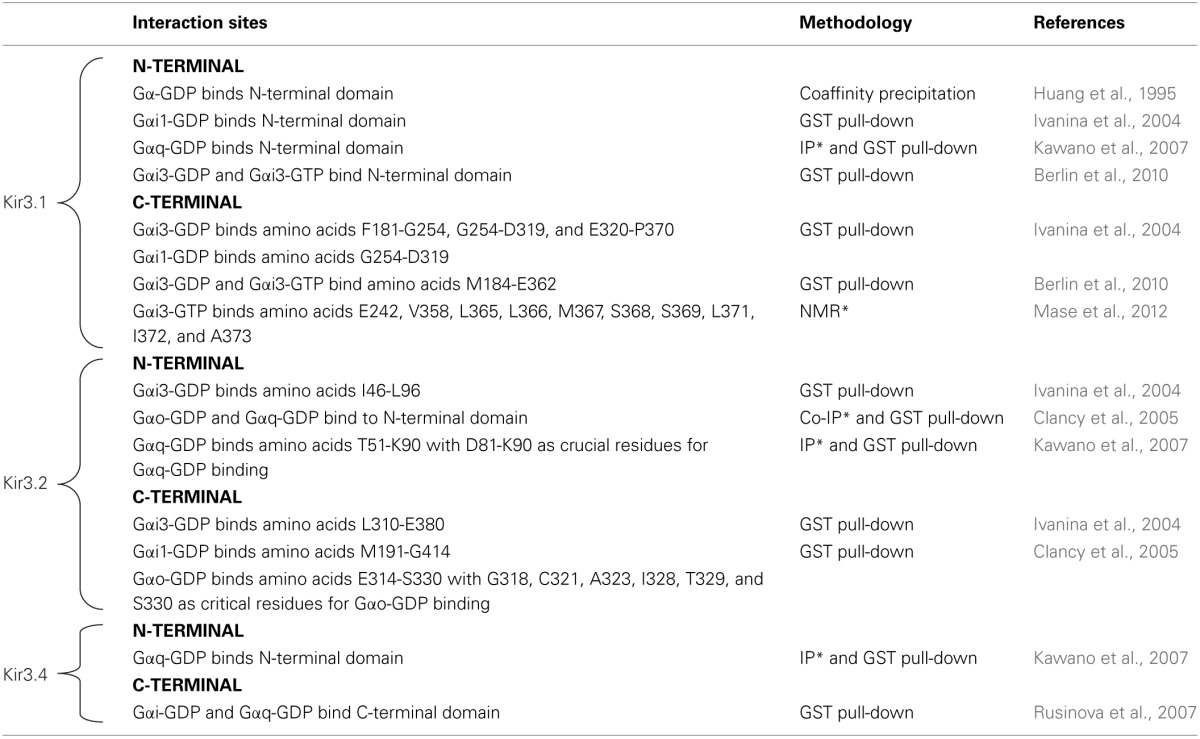
**Gα interaction sites on different Kir3 subunits**.

*IP, Immunoprecipitation; NMR, Nuclear magnetic resonance spectroscopy*.

Although structural determinants of channel activation are matter of considerable consensus, the dynamics that govern the process remain subject of active investigation. Originally, GPCRs, G proteins and channels were conceived as isolated membrane entities capable of conveying pharmacological stimuli from receptors to effectors through a series of collisions which included the G protein as intermediary (Orly and Schramm, 1976; Tolkovsky and Levitzki, 1978). However, the perception that this original formulation of the collision coupling model failed to account for the specificity and temporal resolution of Kir3 channel signaling has led to the proposal of alternative paradigms (Neubig, 1994). One of such alternatives postulates that receptors and their signaling partners may be precoupled in the absence of agonist (Wreggett and De Lean, 1984; Tian et al., 1994) while another proposes that receptors, their G proteins and effectors are compartmentalized within microdomains (Neubig, 1994; Neer, 1995) where signaling partners are present in high enough concentrations to allow rapid interactions by collision (Gross and Lohse, 1991). In the case of neurons, postsynaptic densities are typically specialized domains where anchoring and scaffolding proteins control signaling partners present within dendritic spines (Romero et al., 2011; Fourie et al., 2014). Kir3.2c subunits directly interact with post synaptic density protein 95 (PSD95) and synapse-associated protein 97 (SAP97), two such proteins which not only regulate local concentration of channel subunits but their responsiveness to G proteins as well (Inanobe et al., 1999; Hibino et al., 2000; Nassirpour et al., 2010).

The idea that receptors and channels may associate to form a complex is supported by evidence obtained in native and heterologous systems. Thus, in co-immunoprecipitations from brain samples Kir3 subunits can be recovered with dopamine D_2_ (Lavine et al., 2002) or GABA_B_ receptors (Ciruela et al., 2010). Functional evidence is also consistent with the notion that native receptors and channels may associate. For example, immobilization of MORs receptors expressed in cerebellar granule cells does not alter the rate of Kir3 channel activation, implying that neither species freely diffuses within the membrane during signaling (Lober et al., 2006). More at a systems level, neuroadaptive changes induced by psychostimulants also suggests physical association between receptors and Kir3 channels. In particular, heterologous regulation of GABA_B_ receptors causes them to co-internalize with Kir3 subunits in the ventral tegmental area (VTA) (Padgett et al., 2012) and in pyramidal neurons of the prelimbic cortex (Hearing et al., 2013) following administration of cocaine or amphetamines. Similar conclusions were drawn from studying receptor trafficking in PC12 neurons where homologous desensitization of native muscarinic M2 receptors drives internalization and intracellular accumulation of Kir3 subunits (Clancy et al., 2007). However, a limitation with this group of observations is that native systems not always allow to rule out receptor activation by endogenous ligands, making it difficult to ascertain whether signaling complexes are spontaneously formed or if they result from receptor activation.

Spectroscopic studies in overexpression systems have proven valuable in assessing spontaneous interactions and in providing detailed kinetics of the steps involved in Kir3 activation. In heterologous systems, fluorescence resonance energy transfer (FRET) and bioluminescence resonance energy transfer (BRET)-based approaches have both revealed spontaneous energy transfer between channel subunits and G proteins (Riven et al., 2006; Robitaille et al., 2009; Berlin et al., 2010, 2011) and between the latter and receptors (Rebois et al., 2006; Audet et al., 2008). For opioid receptors in particular, DORs were shown to organize into multimeric arrays that also contain GαoAβ1γ2 and Kir3.1/Kir3.2 subunits (Richard-Lalonde et al., 2013). However, some of these constitutive associations have not been consistently observed. For example, BRET studies show that α2A-adrenergic receptors precouple to Gαi1 (Gales et al., 2006) but FRET data indicate that precoupling occurs with Gαo (Nobles et al., 2005) but not Gαi1 (Hein et al., 2005). These discrepancies have been explained by different arguments: (a) differences in sensitivity between the two techniques would allow BRET to detect lower basal levels of interaction than FRET; (b) receptors do not display the same affinity for different Gα subunits, and (c) receptors with different levels of constitutive activity have different levels of G protein precoupling as reviewed in Lohse et al. (2012).

Kinetic approaches have also addressed the question of whether receptors and G protein precouple in the absence of ligand. For example, FRET assays have established that the time course of conformational changes undergone by the receptor's third intracellular loop upon its activation (50 ms –1 s) (Vilardaga et al., 2003) may be undistinguishable from the kinetics of receptor conformational rearrangements with respect to the G protein (Hein et al., 2005; Jensen et al., 2009). Although these findings argue in favor of precoupling, other observations can be taken as evidence of collision, particularly the fact that the speed of FRET changes that were observed at the interface of the receptor with the G protein varies with the concentration of agonist used to activate the receptor (Hein et al., 2005) and with the amount of G proteins expressed (Hein et al., 2005; Falkenburger et al., 2010). Indeed, both findings are consistent with the essence of the collision model, namely that activated receptors have free access to a common pool of G-proteins. Nonetheless these observations can also be accommodated by a precoupling model if one conceives receptor signaling in terms of conformational ensembles (Kenakin and Onaran, 2002). According to the latter model, a FRET value can be considered representative of a macroscopic state which arises from an ensemble of different receptor states. Within this context, FRET changes corresponding to “receptor activation” represent a multiplicity of states undergoing some degree of conformational change which involves the displacement of the third intracellular loop, not all of which necessarily achieve the full conformational alteration that leads to G protein activation. As agonist concentrations increase and more receptors become permanently occupied by the ligand, the ensemble is progressively constrained so that all states achieve the conformational change that effectively modifies energy transfer between receptors and downstream signaling partners. The increased efficiency, with which higher concentrations of agonist allow the ensemble to attain conformational changes that fully modify the receptor G protein interface, may translate as an increase in the speed with which the two signaling partners reach maximal FRET. This enhanced efficiency to attain full activation may take place in a receptor ensemble that is precoupled to the G protein. Converse reasoning may be applied to explain how an increase in G protein concentrations may enhance the speed of FRET changes at its interface with the receptor in the context of a precoupling model. Indeed, when G proteins are a limiting factor, some receptors are precoupled and others not. As recently demonstrated in crystallographic studies, agonist-occupied receptors will not become fully activated unless coupled to a G protein (Rasmussen et al., 2011). In such case enhanced precoupling that takes place upon higher availability of the G protein will increase the probability of the receptor ensemble of achieving a full active state which evokes an effective conformational rearrangement vis a vis the G protein. As above, the ensemble's improved efficiency to achieve this activation state can be perceived as an increase in the speed with which energy is transferred between activated receptors and G proteins.

FRET technology has also been used in combination with total internal reflection to demonstrate that G protein subunits and Kir3 channels may organize into a membrane-delimited preformed complex (Riven et al., 2006). Constitutive association between Kir3 and Gβγ subunits has also been observed by means of BRET (Rebois et al., 2006; Robitaille et al., 2009; Richard-Lalonde et al., 2013). Moreover, the fact that kinetics of Kir3 channel currents are concordant with conformational changes undergone within the Gαβγ trimer upon activation (Bunemann et al., 2003), has been taken as an additional argument favoring pre-association between G proteins and channel effectors (Lohse et al., 2012).

**Figure 1:**
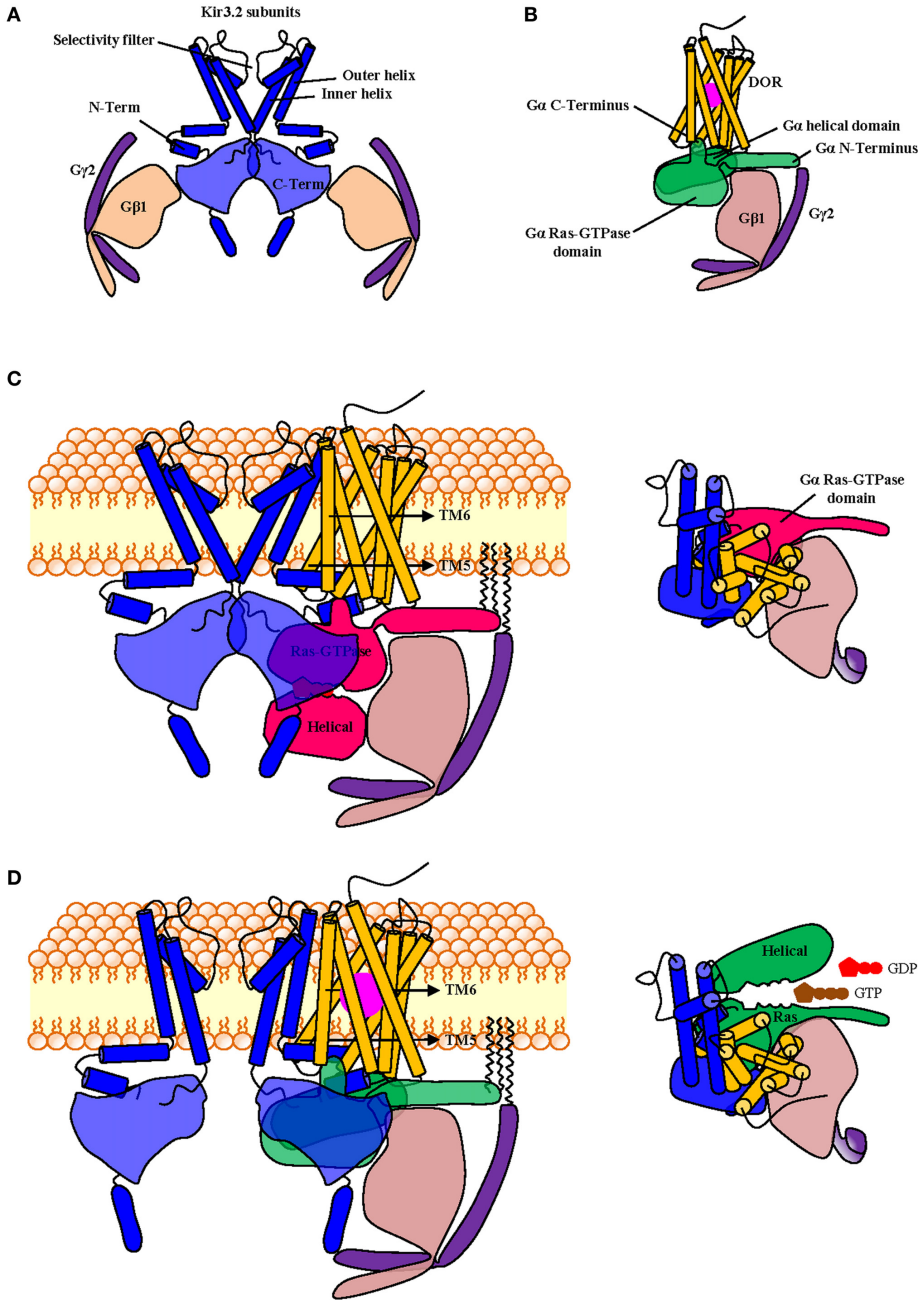
**Schematic representation of putative spatial organization of DORs, Kir3.2 and the Gαβγ subunits within a signaling complex containing all signaling partners**. The complex was constructed from diagrams based on the published crystal structures of the Kir3.2 tetramer in association with corresponding Gβ1γ2 subunits (PDB: 4KFM) **(A)** and that of the nucleotide-free Gαsβ1γ2 trimer in association with the active β 2 adrenergic receptor (PDB: 3sn6) where the latter was replaced by the topography corresponding to the DOR crystal (PDB: 4EJ4). Note that in the active receptor-G protein complex only the Ras-GTPase domain of Gα is fully visible **(B)**. To construct the multimeric array, the DOR-Gαβγ complex shown in B was associated to the Kir3.2 channel shown in **(A)** by superimposing both Gβγ dimers and then removing the one corresponding to the channel. DOR and Kir3.2 subunits were both aligned with respect to the plain of the membrane. By completing this operation transmembrane domains 5 and 6 of the receptor came in close proximity of the outer helix of the channel subunit. The complex is shown in its inactive state where the helical and GTPase domains of Gα are visible and in contact with Gβγ. The inset shows topography of a single channel subunit with its corresponding Gαβγ heterotrimer and DOR seen from above **(C)**. In the active complex the agonist (violet) is bound to the receptor, the third intracellular loop is displaced toward the channel, the C-terminal end of Gα insinuates between intracellular loops 2 and 3. For the proposed multimeric organization to be functional the complex must allow the displacement of the helical domain of Gα upon nucleotide exchange; this is indeed the case since its helical domain moves laterally, from its initial position in the lower part of the inactive complex. Inset shows topography from above **(D)**.

An aspect upon which BRET and FRET data consistently agree, is the fact that G proteins remain associated with the receptor at least during initial stages of signaling (Gales et al., 2005, 2006; Hein et al., 2005, 2006) implying that at one point in time the receptor, the G protein and the effector are all part of the same complex. This reasoning is also in line with evidence summarized in the previous paragraph, which would place the transducer in direct contact with the effector even before activation. This kind of “triple multimeric array” has been described for DORs, GαoAβ 1γ2 and Kir3.1/Kir3.2 subunits using BRET assays and co-immunoprecipitation in overexpression systems. Moreover, BRET changes that were observed among different interaction partners revealed that the conformational information that is codified by agonist binding to the receptor is relayed to the channel via the G protein (Richard-Lalonde et al., 2013). Figure 1 shows a schematic representation of how DORs, Kir3.2 and Gαβγ subunits could organize within a multimeric array with the receptor in its inactive (Figure 1C) or its active (Figure 1D) states. The proposed complex is based on published structures for the Gβγ-bound semi-open Kir3.2 channel (1A) (Whorton and MacKinnon, 2013) and an activated receptor-G protein complex (1B) (Rasmussen et al., 2011). In this putative array receptor and channel were allowed to maintain their positioning with respect to the plain of the membrane while receptor and G protein maintained their relative orientation with respect to one another, as described for the crystallized receptor-G protein complex (Rasmussen et al., 2011). Interestingly, if the diagram had been produced maintaining Gβγ's inclination with respect to the channel (Whorton and MacKinnon, 2013), the latter would have collided with the receptor. This suggest that, in order to organize into a complex, the different signaling partners most likely influence their mutual positions. If each of the four Gβγ subunits, that associate to Kir3.2 subunits in the crystal, interacts with one Gαi/o, and these in turn couple to a corresponding GPCR, it is conceivable that one receptor-G protein complex could occupy one of the grooves that correspond to the site of interaction between two adjacent Kir3 subunits (Figures 1C,D). A supramolecular organization which involves simultaneous association of all signaling partners is compatible with the notion that ligand-specific conformational changes undergone by the receptor can translate into ligand-specific patterns of channel activation. Moreover, allosteric interactions within the array could allow a precoupling model to explain additional observations that are usually attributed to a collision model. For example, the fact that it is possible to attain maximal Kir3 channel currents at concentrations that produce submaximal conformational rearrangement of receptor-G protein interface (Hein et al., 2005) can be explained by positive cooperativity among channel subunits and with the activated Gβ γ dimers, even if not all receptors have been occupied and undergone conformational changes associated with activation.

## Is it possible to bias pharmacological stimuli toward Kir3 channel activation?

Although DORs agonists effectively alleviate chronic pain and have milder side effects than ligands acting at other opioid receptors (Gallantine and Meert, 2005; Feng et al., 2006; Codd et al., 2009), their potential for tolerance (Pradhan et al., 2010; Audet et al., 2012) limits their possible application as therapeutic agents. Given the contribution of cyclase pathway adaptations to the development of this side effect (Javed et al., 2004; He and Whistler, 2007; Bobeck et al., 2014), biasing pharmacological stimulus toward Kir3 channel activation and/or away from cyclase modulation was proposed as a rational means of reducing tolerance. Importantly, together with voltage-gated K^+^ channels (Wimpey and Chavkin, 1991; Moore et al., 1994) and β arrs, Kir3 channels may also participate in hippocampal hyperexcitability (Luscher et al., 1997) induced by certain DOR ligands (Broom et al., 2002; Jutkiewicz et al., 2005). Depending on the degree of Kir3 involvement in this side effect, the incidence of seizures could increase for DOR ligands with Kir3 signaling bias. If this were the case, the alternative strategy based on obliteration of cyclase modulation would be of choice.

*Biased agonism* refers to the ability of orthosteric receptor ligands to selectively engage the activity of a distinct set of signaling partners over another (Urban et al., 2007), a type of selectivity that ensues from the stabilization of receptor conformations which activate a specific effector(s) while sparing the rest (Kenakin and Miller, 2010). Functional selectivity may also be indirect, driven by allosteric ligands (Leach et al., 2007; Kenakin, 2008). Sodium is an allosteric modulator of DORs and manipulation of its binding site provides an example of how allosteric influences may direct pharmacological stimuli toward different effectors (Fenalti et al., 2014). In particular, mutation of one of the residues (Asn131) in the first coordination shell of the Na^+^ ion produced an “efficacy switch” that changed DOR signaling from the canonical Gαi/o pathway toward β arr recruitment. Furthermore, this effect was ligand sensitive since agonists lost Gαi signaling while the antagonist naltrindole gained the ability to recruit β arr (Fenalti et al., 2014). Hence, by stabilizing receptor conformations that differentially favor one orthosteric response over another, *allosteric modulation of receptor conformations* offer a great potential for directing pharmacological stimuli toward a desired response.

Let us first consider signaling bias within the context of the traditional shuttling-collision model. According to this paradigm the agonist interacts with a receptor which then travels within the membrane to interact and activate a G protein whose Gα and Gβ γ subunits subsequently dissociate from the ligand-receptor complex to find and activate an effector (Orly and Schramm, 1976; Tolkovsky and Levitzki, 1978; Gilman, 1987; Bourne, 1997). Because in this model receptor and effector do not simultaneously interact with the G protein, the paradigm does not provide for a “memory” that would allow transferring conformational information codified by the agonist-bound receptor beyond the transducer stage. However, more recent spectroscopic studies of receptor-G protein-effector interaction point to greater restriction in mobility where G protein and effectors would be spontaneously coupled (Lohse et al., 2012). Moreover, independent of whether receptors form part of this constitutive complex or not, evidence analyzed in the previous section indicated that all three species may persistently associate during signaling. This association provides the basis for a “conformational memory” and the possibility of exploring novel bias strategies to specifically direct the pharmacological stimulus of a given receptor (in this case DORs) to a desired G protein coupled effector (Kir3 channels).

**Figure 2:**
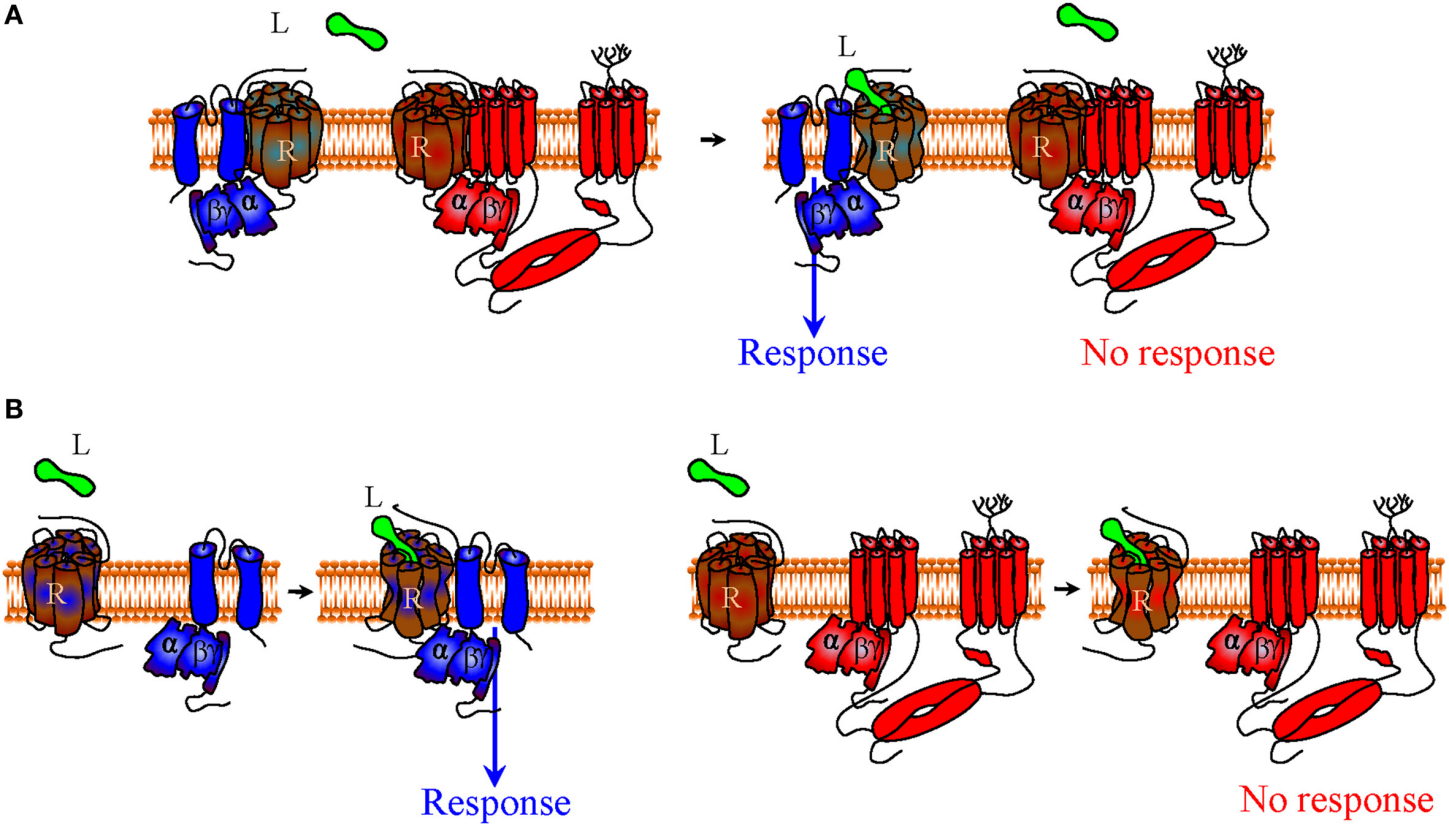
**Functional selectivity due to biased signaling of orthosteric receptor agonists**. Within the context of a precoupling model, an agonist that preferentially recognizes the receptor conformation stabilized by signaling partners in the channel complex over the receptor state stabilized within the cyclase complex would display bias toward channel signaling **(A)**. In a restricted collision model G proteins and effectors coexist as a preformed complex that is activated through collision with the agonist-bound receptor. In this context bias toward Kir3 signaling can take place if the agonist-bound receptor displays higher affinity for Gαβγ-Kir3 than Gαβγ-cyclase complexes **(B)**. To aid visualization, Kir3 channel is shown in blue and adenylyl cyclase in red.

Being allosteric proteins (Kenakin and Miller, 2010), the conformation adopted by the receptor will not be solely determined by orthosteric agonist binding, but also by its interaction with its cytosolic (G protein subunits) and membrane (Kir3 or cyclase) signaling partners, which therefore function as natural allosteric modulators. In cases where signaling complexes exist before activation, selectivity favoring Kir3 vs. cyclase signaling could be achieved by designing orthosteric ligands that display higher affinity for the conformation adopted by the receptor when contained within a Kir3 signaling complex than the one induced by its inclusion into cyclase multimers (Figure 2A). The idea that orthosteric agonists can indeed be tailored to specifically recognize receptors in association with distinct signaling partners is supported by studies indicating that the pharmacological properties of receptors contained in homodimers are different from those displayed by the same receptor when it is part of an heterodimer (Jordan and Devi, 1999; Waldhoer et al., 2005). Alternatively, if the complex is formed during signaling, bias toward Kir3 channel effectors would depend on the agonist's ability to stabilize a receptor conformation whose affinity for the G protein/Kir3 complex is higher than the one displayed for the G protein/cyclase complex (Figure 2B).

**Figure 3:**
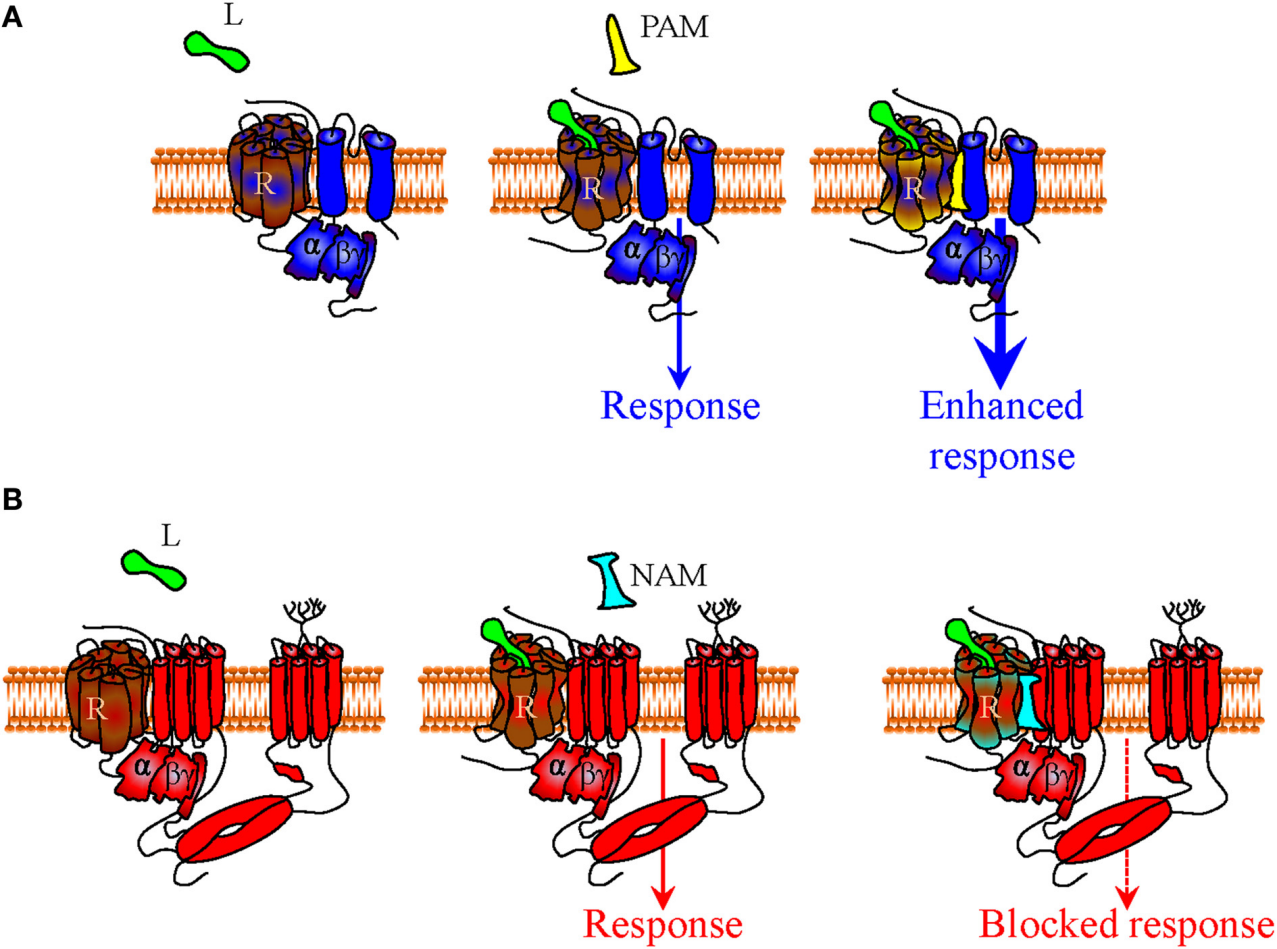
**Mechanisms of indirect bias involving allosteric modulators that specifically recognize a complex of desired composition**. In a precoupling model or in a restricted collision model receptors, G proteins and effectors may all persistently associate during signaling. Within this context (only precoupling model represented in figure), small positive allosteric modulators (PAM) that specifically recognize the interface between DORs and Kir3 channels (in blue) may specifically bias signaling of DOR orthosteric agonists toward this effector by stabilizing the complex and/or favoring channel opening **(A)**. A negative allosteric modulator (NAM) that recognizes the DOR-adenylyl cyclase (in red) interface could distinctively block cAMP inhibition by the activated DOR, also allowing for bias in favor of channel effectors **(B)**.

**Figure 4:**
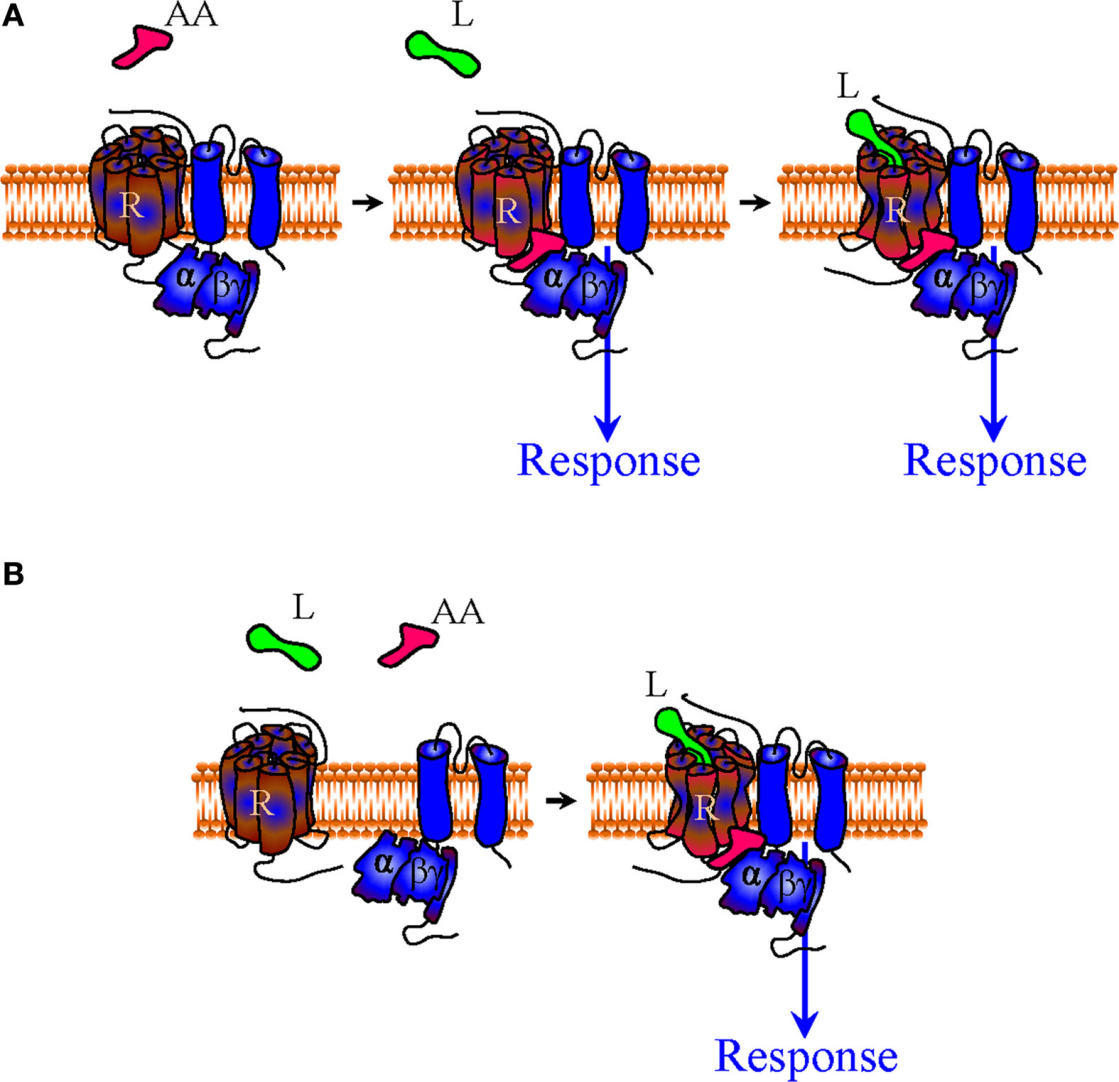
**Mechanisms of indirect bias involving allosteric agonists that specifically recognize a complex of desired composition**. A complex-specific allosteric agonist (AA) may distinctively recognize an interface that is unique to the complex of interest. In the context of the precoupling model AA may initiate signaling independent of whether the orthosteric agonist is present or not **(A)**. In the restricted collision model the presence of the orthosteric agonist is necessary for the complex to be formed and provide the binding site for AA **(B)**.

In addition, if at rest and/or during signaling receptors, G proteins and effectors associate through a distinct network of conformational influences, it is also conceivable that the pharmacological stimulus that is produced by an orthosteric ligand could be influenced by small *allosteric modulators* that specifically *recognize the complex* with the desired combination of DORs, Gαβγ subunits and Kir3 channels. For example, small molecules that could bind the interface of DORs and Kir3 subunits to stabilize the complex and/or favor channel opening (*positive allosteric modulators*), would be of particular interest since they could selectively enhance Kir3 signaling by DORs and no other receptors that modulate this effector (Figure 3A). Alternatively, a *negative allosteric modulator* that recognizes the DOR/adenylyl cyclase interface could distinctively block cAMP inhibition by this receptor, resulting in another desired type of bias (Figure 3B). Finally, a variation of this strategy would be to design *complex-selective allosteric agonists* which are able to initiate signaling, independent of whether the orthosteric ligand is present or not (Figure 4A), or only when it is present for the case of restricted collision (Figure 4B). Such type of ligand could putatively recognize and stabilize the interface formed between the C-terminus of the activated Gαi/o subunit, the channel N-terminus and the third intracellular loop of the receptor. Admittedly, the structural information required for designing these compounds is not yet available but should become available once receptor/G proteins complexes are co-crystallized with their effectors.

In summary, we have analyzed evidence indicating that Kir3 channels are mediators of opioid analgesia. While they play a considerable role in undesired effects of MOR agonists, their contribution to those of DOR ligands is limited. Biasing DOR responses in favor of Kir3 channels and away from cyclase inhibition was suggested as a means of controlling analgesic tolerance of DOR agonists, a side effect that limits their potential therapeutic application. Different modalities of GPCR association with G proteins and effectors were discussed, and putative bias strategies to ensure specific activation of a desired combination of receptors (DORs) and effectors (Kir3 channels) were provided.

### Conflict of interest statement

The authors declare that the research was conducted in the absence of any commercial or financial relationships that could be construed as a potential conflict of interest.

## Abbreviations

GIRK: G protein activated inward rectifier K^+^
GPCRs: G protein-coupled receptors
DORs: Delta opioid receptors
MORs: Mu opioid receptors
KORs: Kappa opioid receptors
PLC: Phospholipase C
GABA: Gamma-aminobutyric acid
DRG: dorsal root ganglion
DAMGO: [D-Ala(2),N-Me-Phe(4),Gly(5)-ol]-enkephalin
KCNJ6: Inwardly-rectifying potassium channel, subfamily J, member 6
KCNJ3: Inwardly-rectifying potassium channel, subfamily J, member 3
5-HT: 5-hydroxytryptamine
MAPK: Mitogen-activated protein kinases
βarr: βarrestin
cAMP: Cyclic adenosine monophosphate
VTA: Ventral tegmental area
PIP2: Phosphatidylinositol 4,5-bisphosphate
GDP: Guanosine diphosphate
GTP: Guanosine triphosphate
PSD95: Postsynaptic density protein 95
SAP97: Synapse-associated protein 97
FRET: Fluorescence resonance energy transfer
BRET: Bioluminescence resonance energy transfer
PC12: Neuron-like pheochromocytoma.
